# Editorial: Mind over brain, brain over mind: cognitive causes and consequences of controlling brain activity - volume II

**DOI:** 10.3389/fnhum.2023.1280095

**Published:** 2023-09-21

**Authors:** Elisabeth V. C. Friedrich, Christa Neuper, Reinhold Scherer

**Affiliations:** ^1^Research Unit Biological Psychology, Department of Psychology, Ludwig-Maximilians-Universität München, Munich, Germany; ^2^Neuropsychology, Department of Psychology, University of Graz, Graz, Austria; ^3^Brain-Computer Interfaces and Neural Engineering Laboratory, School of Computer Science and Electronic Engineering, University of Essex, Colchester, United Kingdom

**Keywords:** brain-computer interface (BCI), neurofeedback (NF), neuro-rehabilitation, multisensory feedback, cognitive improvement, advances and challenges, translation

One of the central neuroscientific questions we have been trying to answer for years is that of the causal relationships between mental activity (i.e., cognitive processes), brain signals (i.e., their physical correlate), and the ability to control a brain-computer interface (BCI) or benefit from neurofeedback (NF) interventions. To get closer to an answer, this question was divided into smaller and more manageable challenges: can a person's ability to modulate brain signals provide information about how the brain implements cognitive processes? In turn, can these processes determine how well one can control one's own brain signals? Does the ability to switch rapidly between mental activities or brain states (i.e., to increase/decrease signal amplitudes in a specific frequency band over a particular brain area) lead to lasting changes in cognitive abilities?

Machine learning-assisted neurotechnology detects patterns in neural signals modulated by different cognitive tasks and converts these distinct patterns into information for communication and control (e.g., by means of a BCI). In addition, learning to modulate one's own brain activity can lead to beneficial changes in cognition and behavior, which is used for therapeutic goals (e.g., NF). However, the correspondence between brain signals and cognitive activity is difficult to capture because of the high complexity and nonlinear behavior of neural and biological systems, the interindividual differences, and the changes in neural signal properties over time. Moreover, the brain generates a large amount of coherent spontaneous activity regardless of the task at hand, which can be detrimental to the generation of distinguishable patterns from brain signals. This is especially true for non-invasive methods that measure the sum activity of a large number of neurons. As a result, it is difficult to identify clear patterns in many people, which currently limits the potential of neurotechnology. We addressed these problems a decade ago in our first Research Topic (Friedrich et al., 2014). Now it is time for an update.

In the last ten years, further technological and methodological advances have been made and neuroscientific knowledge gained, leading to significant performance increases in BCI. Specifically, when using invasive recording approaches and employing deep artificial neural networks for pattern recognition. For example, speech neuroprostheses can reliably decode individual letters or full words from electrocorticography (Metzger et al., 2022). An overview of the current state of the art of methods developed or applied for the classification of cognitive and motor tasks from intracranial recordings was provided by Mirchi et al.. Kennedy and Cervantes have shown that single neurons not associated with a specific task can be recruited and conditioned to fire when given appropriate task-related feedback. While individual neurons have specific functions, the authors conclude that recruitment of additional neurons has the potential to expand the range of the population of recorded neurons and improve the stability of neural patterns and thus the reliability of pattern recognition for BCI.

In the field of non-invasive BCI and NF, technological progress has been used to improve the user experience and enable new multisensory feedback. Virtual Reality and neurotechnology were combined to implement more holistic experiences for neuro-rehabilitation. Pais-Vieira et al. combined visual, auditory, tactile, and thermal feedback to create embodiment experiences and relieve chronic pain in a patient with spinal cord injury.

There have also been new developments in EEG-based NF training (NFT). Kleih-Dahms and Botrel implemented a slow cortical potential NFT to improve chronic attention deficits after stroke. In a case-by-case analysis, the findings suggest that NFT has a positive impact on post-stroke cognitive deficits and quality of life. Smit et al. investigated the usefulness of NFT to achieve cognitive improvements in subclinical participants. The NFT aimed at increasing frontal midline theta activity in order to improve subjective experienced problems in tasks relying on executive functions in daily life.

Both studies illustrate that the problem of interindividual differences, non-responders, and the high time investment required for training is still an important issue. Another challenge that remains is the translation of BCIs or NF to real-world applications such as home use of NFT. Autenrieth et al. reported that in an attempt to implement home-based NFT with minimal remote expert support, more than half of the participants dropped out. The remaining participants failed to learn how to modulate their SMR activity within nine sessions.

The often-envisioned general BCI or NF solution “for all” has not yet been realized. Given the many challenges mentioned in the introduction, the considerable interindividual variability of brain signals and inaccuracies in the assessment of mental activity, this would also have been very surprising. It still appears that BCI model parameters should be as individualized and adaptable as possible to provide each individual with the optimal opportunity to control a device (Friedrich et al., 2013; Faller et al., 2014; Scherer et al., 2015; Cunha et al., 2021). Unlike BCIs, NFTs are often hypothesis-driven, i.e., based on the assumption that different properties in brain signals across specific brain regions are related to different cognitive processes and their impairments. A way forward seems to combine this theory-driven approach with the empirical approach and examine individual brain activity to test whether the hypothesis holds for the individual (Friedrich, 2020). A systematic investigation of baseline (i.e., pre-BCI/NFT) differences between responders and non-responders should shed light on why it works for some but not all participants. Since feedback is essential to NF, the use of relevant feedback (i.e., feedback directly corresponding to the brain signal/region being trained and to the targeted cognitive/motor process) (Friedrich et al., 2015) or holistic feedback (Pais-Vieira et al.) has the potential to significantly improve performance/therapeutic goals. In addition, we should not only consider the specific target brain signal or region, but also look at the entire activity network and communication between brain regions.

BCI and NF approaches present significant neuroscience and technology challenges, but also hold enormous potential to improve people's lives. Therefore, it is our commitment to further research and develop these technologies so that as many people as possible can use them for various applications and they become useful solutions that work also outside of controlled laboratory environments.

## Author contributions

EF: Conceptualization, Writing—original draft, Writing—review and editing. RS: Conceptualization, Writing—original draft, Writing—review and editing. CN: Conceptualization, Writing—review and editing.
